# Self-Identified Age Cohorts and Personal Experience Sharing in Pseudonymous Online Spaces: Natural Language Processing Analysis of Reddit

**DOI:** 10.2196/90103

**Published:** 2026-05-12

**Authors:** Ern Chern Khor, Moon Choi

**Affiliations:** 1 Graduate School of Science and Technology Policy Korea Advanced Institute of Science and Technology Daejeon, Daejeon Republic of Korea; 2 Graduate School of Data Science Korea Advanced Institute of Science and Technology Daejeon, Daejeon Republic of Korea

**Keywords:** older adults, age cohort, life course, social media, Reddit, pseudonymity, self-identification, self-disclosure, natural language processing, machine learning, social support, digital inclusion

## Abstract

**Background:**

While older adults’ social media use has been widely studied for its instrumental benefits, such as accessing health information or maintaining family ties, research has largely focused on identity-based platforms that mirror offline social networks, leaving pseudonymous, interest-driven environments such as Reddit underexplored. Although older adults actively participate in these spaces to share personal narratives and engage beyond their existing social roles, the literature has yet to center their own voices, with most existing work focusing on caregivers or younger users discussing older adults rather than older adults speaking for themselves.

**Objective:**

Integrating a life course perspective with the concept of subjective age cohort, this study examines how Reddit users who explicitly self-identify as being in their 50s, 60s, or 70s engage in self-expression and personal experience sharing within pseudonymous online spaces.

**Methods:**

Using data collected via the Reddit application programming interface, this study analyzed posts and comments from 848 self-identified older Reddit users, including 488 future older adults in their 50s (57.5%) and 360 current older adults in their 60s and 70s (42.5%) identified through age-based flairs in the subreddit *r/AskOldPeople*. A fine-tuned large language model (BLOOM-560m; *F*_1_-score=0.96) classified 4,055,275 sentences into three personal experience domains: (1) health and wellness, (2) personal relationships and identity, and (3) professional and financial life. Chi-square analysis compared domain distributions across age groups. BERTopic topic modeling identified thematic patterns within each domain.

**Results:**

Of 569,107 personal experience sentences identified, personal relationships and identity comprised the largest share (n=268,212, 47.1%), followed by professional and financial (n=186,768, 32.8%) and health and wellness (n=114,127, 20.1%). Chi-square analysis revealed significant between-group differences (*χ*^2^_2_=34.7; *P*<.001): current older adults shared proportionally more about health and wellness, whereas future older adults shared more about relationships and identity. Topic modeling further revealed qualitatively distinct emphases within each domain. Future older adults’ posts frequently discussed menopause, depression, and friendships, while current older adults more frequently addressed chronic illness, aging, and financial security. Both groups used pseudonymity to disclose sensitive or stigmatized topics.

**Conclusions:**

This study demonstrates that older adults’ personal experience sharing on pseudonymous platforms is shaped by the simultaneous interplay of life course stage, age cohort background, and platform affordances, rather than by any single factor. Pseudonymous environments such as Reddit enable disclosures of health vulnerabilities, contested social identities, and emotional experiences that are structurally discouraged on identity-based networks, and this has direct implications for how digital inclusion is conceptualized and practiced. Beyond operational literacy, supporting older adults’ expressive inclusion in interest-based, pseudonymous online communities represents a meaningful yet underaddressed dimension of digital participation policy.

## Introduction

### Background and Research Gap

Older adults’ use of social media is often examined through the lens of instrumental benefits, such as accessing health-related information or maintaining contact with family and friends [1-3]. Yet, this perspective largely centers on identity-based platforms that replicate offline social networks. A growing body of research distinguishes between platforms that sustain identity-based networks, such as Facebook or Instagram, and those that are pseudonymous and interest-based, such as Reddit (Reddit, Inc), where users connect with strangers through shared topics rather than preexisting ties [4,5]. Unlike social networking sites grounded in real-name connections, these interest-driven environments encourage interaction that is less constrained by existing social roles and expectations.

Despite growing recognition of older adults’ digital participation, a critical gap remains in the literature: a distinct lack of research that prioritizes older adults’ own voices and personal sharing within pseudonymous online environments. While some studies have examined aging-related discussions on these platforms, most have focused on caregivers or younger users talking about older adults [6,7]. Given that older adults actively participate in various online environments to learn from and interact with younger generations and to share their own narratives and reflections [8,9], such pseudonymous spaces allowing users to interact free from existing offline social roles may therefore provide meaningful yet overlooked contexts for older adults to communicate and sustain social contact in digital spaces. This study addresses this gap by shifting the focus to older adults’ self-expression on Reddit, examining how they navigate and share personal experiences when untethered from identity-based networks.

### Theoretical Framework

From a social relations perspective, the convoy model provides a rationale for why such spaces can matter in later life [10,11]. As individuals age, their social networks, or convoys, tend to shrink due to retirement, mobility limitations, or loss of peers. Supplementary, low-barrier online exchanges can partially compensate for this contraction by maintaining a sense of embeddedness and continuity. While the convoy model explains why older adults may be drawn to online pseudonymous platforms, a life course perspective is needed to understand how they engage with them. In this perspective, age is understood not only as biological age but also as a series of transitional life phases [12]. Rather than viewing age as fixed, the life course approach conceptualizes it as a dynamic process in which each stage is shaped by distinct social and developmental characteristics [13]. These age-related characteristics influence media use alongside changing social circumstances [14]. Unlike rigid biological age, the life course perspective attempts to capture the social dynamics of individuals in relation to their broader societal context [15]. An individual’s current life stage, such as transitioning out of the workforce, brings specific social conditions that can influence the types of experiences they wish to discuss with others.

### Self-Identification on Pseudonymous Platforms

In pseudonymous spaces like Reddit, the intersection of life course and age cohort becomes highly visible through self-identification. Because Reddit relies on anonymity, a user’s age group is invisible unless they explicitly disclose it. This act of voluntary disclosure can be understood through the lens of subjective age identification, which examines the age group to which a person self-identifies, regardless of their strict chronological age [13]. Often referred to as “identity age,” this self-categorization grounds age at a subjectively experienced level rather than an objective one [13]. In this sense, one’s age cohort self-identified on Reddit signals the social realities of their life course stage.

Within this integrated framework, pseudonymous, topic-based platforms such as Reddit offer an important setting for examining how these identities are expressed in practice. Their affordances, namely, default anonymity, topic segmentation, and interest-based exchange, enable older adults to share personal experiences outside the boundaries of identity-based networks, creating new conditions under which age-group–related patterns of sharing can be reconsidered [16]. On Reddit, users who explicitly self-identify as “60-something” or “70-something” publicly articulate a sense of belonging to an age cohort within a pseudonymous environment. This study focuses on people who consciously identify with these groups and make that identity visible online. Such acts of self-identification involve 2 complementary processes: *individuation*, through which members recognize common traits and shared experiences within the group, and *differentiation*, through which they mark their distinction from other groups [9,17].

### Research Objective

The primary objective of this study is to examine how older adults engage in self-expression and personal experience sharing in pseudonymous online spaces. Rather than treating older adults as a monolithic age group, this study moves beyond demographic categories by integrating a life course perspective with the concept of subjective age cohort. Specifically, it investigates how Reddit users who explicitly self-identify as being in their 50s, 60s, or 70s share personal experiences online. Focusing on users who make their age cohort membership publicly visible, this study interprets such disclosures as performative expressions of subjective age cohort identity. This framing illuminates how people navigating the later stages of midlife and early older adulthood draw on pseudonymous platforms to adapt their modes of self-expression to the particular demands and transitions of their current life stage.

## Methods

### Platform Context

This study adopted a mixed methods approach combining large-scale computational text analysis, statistical comparisons, and topic modeling. The analysis focused on data from Reddit users who explicitly self-identify as being in their 50s, 60s, or 70s. Reddit is a large-scale, forum-based platform where users post and comment within topic-specific communities called *subreddits*. Each subreddit is user-created and moderated, with its own rules, culture, and norms. Users can join and participate in multiple subreddits, allowing diverse discussions across interests and experiences. As of 2024, Reddit ranked as the ninth-most-used online platform in the United States [18]. Unlike identity-based social networks, Reddit operates under pseudonymity, where users participate under consistent usernames that are not tied to real-world identities. This allows them to maintain a recognizable online presence while preserving privacy [19]. Subreddits also allow the use of user flairs, which are short labels that appear beside usernames within a given subreddit. These flairs can indicate characteristics such as age group (eg, “60-something”), experience level (eg, “newbie”), or location (eg, “NYC”).

### Data Collection

This study focused on *r/AskOldPeople*, a subreddit with a strong presence of older adults that facilitates intergenerational and peer discussions about aging and everyday life. As of June 2025, *r/AskOldPeople* had more than 800,000 members and ranked within the top 1% of all subreddits by size. To identify users’ age groups in this pseudonymous environment, the study used the subreddit’s flair system. In *r/AskOldPeople*, users may select flairs such as “50-something” or “70-something” to indicate their age. Data were collected via the Reddit Developer Platform application programming interface (API) in January 2024, retrieving users who posted or commented on the 1000 most recent posts, reflecting the limit imposed by Reddit’s API. Although this constrained the historical range of data, it ensured the inclusion of recently active users with up-to-date age flairs and minimized overrepresentation of highly active users. A total of 848 users were identified for analysis: 360 (42.5%) current older adults (“60-something” and “70-something” flairs) and 488 (57.5%) future older adults (“50-something”). Of the 360 current older adults, 304 (84.4%) were in their 60s, and 56 (15.6%) were in their 70s. These 2 age groups were combined into a single category to ensure a comparable sample size with the future older adults’ group.

Although *r/AskOldPeople* was used to identify older Reddit users, the analysis was not limited to that subreddit. Reddit’s structure allows public posts and comments across all subreddits to be retrieved for a given user. Using the Reddit API in February 2024, up to 1000 of the most recent posts and 1000 of the most recent comments were collected for each identified user. To ensure comparability across varying content lengths, all text was tokenized into sentences using the Punkt sentence tokenizer, which detects sentence boundaries in natural language text. This process yielded a dataset of 4,055,275 sentences: 1,671,400 (41.2%) from current older adults and 2,383,875 (58.8%) from future older adults.

### Personal Sharing Classification

Given the dataset’s scale, a machine learning model was fine-tuned to classify each sentence into one of four categories: (1) health and wellness, (2) personal relationships and identity, (3) professional and financial, or (4) not related to personal experience sharing. After excluding the sentences unrelated to personal experience sharing, only the first 3 categories were analyzed. The classification schema was adapted from Geetha et al [20] and refined to include only personally reflective sentences. When sentences referenced multiple domains, the dominant theme was selected. For example, “My wife was diagnosed with stage IV cancer” was classified as *health and wellness*, while “I kept talking to my therapist when I started dating again after I divorced” was classified as *personal relationships and identity*. Sentences not describing personal experiences (eg, “The International Lyme and Associated Diseases Society recommends 4-6 weeks of antibiotics” or “Thank you so much”) were excluded.

The model used was BLOOM-560m [21], an open-source large language model available via Hugging Face. A balanced training set of 2000 manually labeled sentences (500 per category) was constructed through stratified random sampling and split into training (80%), validation (10%), and test (10%) subsets. The fine-tuned model achieved an *F*_1_-score of 0.96 on the test set and was subsequently applied to label the full dataset. The fine-tuned model is publicly accessible at [22].

### Ethical Considerations

This study used publicly available data collected via the official Reddit API. Data collection complied with the platform’s Terms of Service, and the study qualified for an Institutional Review Board exemption due to the lack of direct human subject interaction. Any direct quotes used for illustration were carefully paraphrased to prevent reverse searches.

### Analytic Strategy

To examine age cohort differences in personal sharing, the proportions for each domain were calculated for both groups. A chi-square test was performed to assess whether the distribution of domains differed between age cohorts, with Bonferroni-adjusted post hoc comparisons conducted when significant.

**Figure 1:**
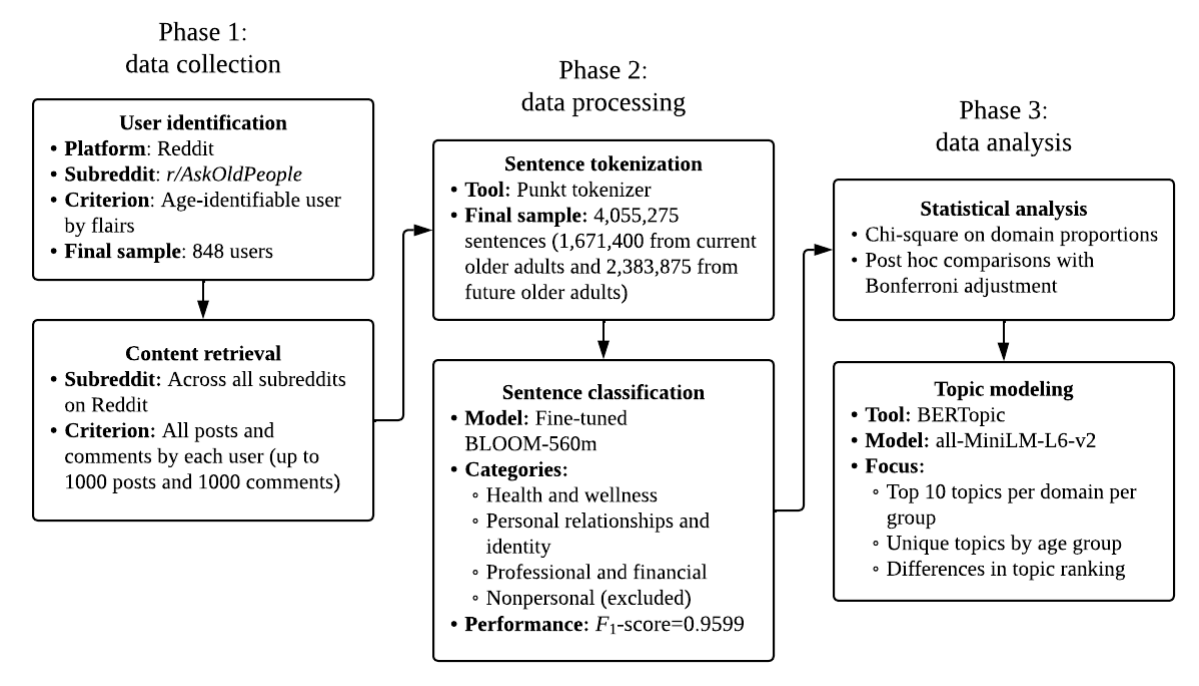
Data collection and analysis process.

To further explore thematic variation within each domain, topic modeling was applied using BERTopic, which uses transformer-based embeddings to cluster semantically related sentences into interpretable themes [23]. The SentenceTransformer model *all-MiniLM-L6-v2* was used to generate embeddings. Topic modeling was conducted separately for each domain and group. For each group, the top 10 topics were identified by frequency, represented by characteristic keywords automatically extracted by the model. The analysis focused on identifying topics unique to one group and differences in topic prominence between current and future older adults. This approach enabled a detailed comparison of how distinct groups of older adults share personal experiences across life domains in pseudonymous online contexts. A visual summary of the data processing and analysis workflow is provided in Figure 1.

## Results

### Overview of Personal Experience Sentences

After excluding nonpersonal content, a total of 569,107 sentences were identified as related to personal experiences. Among these, the “personal relationships and identity” domain accounted for the largest proportion (n=268,212, 47.1%), followed by the “professional and financial” (n=186,768, 32.8%) and “health and wellness” (n=114,127, 20.1%) domains.

### Chi-Square Analysis of Domain Distribution

A chi-square test revealed a significant difference in the distribution of domains between future and current older adults (*χ*^2^_2_=34.7; *P*<.001), with Bonferroni-adjusted post hoc comparisons indicating that the professional and financial domain did not differ significantly between groups. Current older adults in their 60s and 70s shared proportionally more about health and wellness (46,379/227,458, 20.4%; *P*<.001), whereas future older adults in their 50s shared more about personal relationships and identity (161,925/341,649, 47.4%; *P*<.001). Table 1 summarizes the domain distribution and standardized residuals.

**Table 1:** Distribution of personal experience domains by age group.

Age group	Health and wellness		Personal relationships and identity		Professional and financial	
	Values, n (%)	*R* ^a^	Values, n (%)	*R*	Values, n (%)	*R*
Future older adults (50s; n=341,649)	67,748 (19.8)	–5.17^b^	161,925 (47.4)	4.94^b^	111,976 (32.8)	–0.84
Current older adults (60s and 70s; n=227,458)	46,379 (20.4)	5.17^b^	106,287 (46.7)	–4.94^b^	74,792 (32.9)	0.84
Total (n=569,107)	114,127 (20.1)	—^c^	268,212 (47.1)	—	186,768 (32.8)	—

^a^*R* represents standardized residuals from the chi-square test.

^b^Residuals with Bonferroni-adjusted significance levels *P*<.001.

^c^Standardized residuals are not applicable to marginal totals.

### Thematic Patterns by Domain

#### Health and Wellness

Table 2 presents the 10 most frequent topics by domain and age group. In the health and wellness domain, leading themes for future older adults included *smoking and nicotine use* (615/41,134, 1.5%), *cancer experiences* (460/41,134, 1.1%), *hormonal changes* (335/41,134, 0.8%), mental health discussions, such as *depression* (326/41,134, 0.8%), and *health care access* (280/41,134, 0.7%). Sentences commonly described experiences such as treatment side effects (“The estrogen patch stopped my hot flashes”) or emotional responses to illness (“I am much more lonely now and prone to depression”).

For current older adults, the most frequent themes were *smoking* (409/28,376, 1.4%), *keto diet* (365/28,376, 1.3%), *positive health reflections* (296/28,376, 1.0%), *vaccination* (254/28,376, 0.9%), and *aging awareness* (206/28,376, 0.7%). Additional frequent topics included *dental issues* (195/28,376, 0.7%) and *Parkinson’s disease* (190/28,376, 0.7%). Example sentences referenced daily management of age-related conditions (“As I get older, I feel tired and discouraged”) and lifestyle adjustments (“I transitioned from the Standard American Diet to keto gradually”). While some topics overlapped (eg, smoking, vaccination, and cancer), their relative prominence varied between groups.

#### Personal Relationships and Identity

Both groups frequently discussed hobbies and everyday interests, but the emphasis of the discussion differed. For future older adults, top-ranked topics were *biking* (604/83,150, 0.7%), *racial identity* (527/83,150, 0.6%), *LGBTQ+ identity* (445/83,150, 0.5%), *photography* (419/83,150, 0.5%), and *books and reading* (383/83,150, 0.5%). Additional themes included *hairstyles* (353/83,150, 0.4%), *friendship* (302/83,150, 0.4%), and *movies* (283/83,150, 0.3%). Sentences often included midlife reflections on friendships (“I have five friends in my inner circle, and it’s all I need”).

Current older adults similarly mentioned *biking* (597/62,124, 1.0%) and *reading* (364/62,124, 0.6%), but also featured *gun ownership* (232/62,124, 0.4%) and *cooking* (230/62,124, 0.4%) among top topics. Examples included sharing recipes or memories of learning to cook for family (“I’m putting together a cookbook for my sons”). Across both groups, discussions of racial and LGBTQ+ identities were frequent, indicating engagement with broader conversations about social identity on the platform.

#### Professional and Financial Life

Work, income, and retirement were common themes for both groups, though with different foci. Leading topics for future older adults included *ticketing and consumer transactions* (345/63,052, 0.5%), *legal issues* (302/63,052, 0.5%), *work attire* (263/63,052, 0.4%), *retirement planning* (246/63,052, 0.4%), and *tipping practices* (238/63,052, 0.4%). Additional topics involved *job interviews* (205/63,052, 0.3%) and *COVID-related work disruptions* (204/63,052, 0.3%). Example sentences included practical reflections on everyday financial activity and employment: “I already have my tickets for next year,” “I’m a big tipper, so I usually go closer to 30 percent,” and “I have not gotten one single interview this year.”

For current older adults, frequent topics were *legal matters* (326/42,626, 0.8%), *retirement* (312/42,626, 0.7%), *401(k) and pension savings* (241/42,626, 0.6%), *social security* (193/42,626, 0.5%), and *charitable giving* (181/42,626, 0.4%). Other topics included *gender at work* (157/42,626, 0.4%) and *air travel* (148/42,626, 0.3%). Example sentences included: “I am 67, retired in August, and most basic expenses are covered by Social Security and a pension,” “I donate to charities every year,” and “I used to fly weekly for work for many years.” Overall, both groups contributed extensive discourse around financial security and employment experiences, though the specific subthemes differed in prominence.

Across domains, both current and future older users produced diverse personal narratives on Reddit. The largest quantitative differences appeared in the health and wellness and personal relationships and identity domains, whereas professional and financial content showed similar proportions. Topic modeling further revealed that overlapping themes, such as health conditions, hobbies, and work, were framed through distinct emphases in frequency and rank, reflecting the breadth of personal sharing among older Reddit users.

**Table 2:** Top topics by personal experience domain and age group with representative keywords.

Category and topic rank			Future older adults (50s)					Current older adults (60s and 70s)		
			Topic keywords (topic ID)		Value, n (%)^a^		Topic keywords (topic ID)			Value, n (%)
**Health and wellness**										
	Top 1	“Smoking,” “nicotine,” “cigarettes” (T0)		615 (1.5)		“Smoking,” “nicotine,” “cigarettes” (T0)			409 (1.4)	
	Top 2	“Cancer,” “breast,” “stage” (T2)		460 (1.1)		“Keto,” “carb,” “recipes” (T5)			365 (1.3)	
	Top 3	“Estrogen,” “hormone,” “progesterone” (T10)		335 (0.8)		“Healthy,” “thanking,” “happiness” (T1)			296 (1.0)	
	Top 4	“Healthy,” “thanking,” “happiness” (T1)		327 (0.8)		“Vaccine,” “vaccines,” “vaccinated” (T3)			254 (0.9)	
	Top 5	“Depression,” “depressed,” “depressive” (T6)		326 (0.8)		“Aging,” “age,” “old” (T12)			206 (0.7)	
	Top 6	“Health care,” “insurance,” “universal” (T4)		280 (0.7)		“Health care,” “insurance,” “universal” (T4)			200 (0.7)	
	Top 7	“Nurses,” “nurse,” “nursing” (T7)		276 (0.7)		“Dentists,” “dental,” “teeth” (T9)			195 (0.7)	
	Top 8	“Vaccine,” “vaccines,” “vaccinated” (T3)		271 (0.7)		“Parkinson disease,” “cardinal,” “disease” (T55)			190 (0.7)	
	Top 9	“Diagnosed,” “officially,” “rediagnosed” (T8)		242 (0.6)		“Fasting,” “intermittent,” “fasts” (T29)			183 (0.6)	
	Top 10	“Keto,” “carb,” “recipes” (T5)		238 (0.6)		“Cancer,” “breast,” “stage” (T2)			176 (0.6)	
**Personal relationships and identity**										
	Top 1	“Bike,” “bikes,” “ride” (T0)		604 (0.7)		“Bike,” “bikes,” “ride” (T0)			597 (1.0)	
	Top 2	“White,” “racist,” “black” (T1)		527 (0.6)		“Book,” “read,” “library” (T3)			364 (0.6)	
	Top 3	“Gay,” “straight,” “LGBTQ” (T2)		445 (0.5)		“White,” “racist,” “black” (T1)			363 (0.6)	
	Top 4	“Photos,” “photo,” “pictures” (T4)		419 (0.5)		“Gay,” “straight,” “LGBTQ” (T2)			309 (0.5)	
	Top 5	“Book,” “read,” “library” (T3)		383 (0.5)		“Photos,” “photo,” “pictures” (T4)			286 (0.5)	
	Top 6	“Hair,” “curly,” “bangs” (T5)		353 (0.4)		“Hair,” “curly,” “bangs” (T5)			280 (0.5)	
	Top 7	“Blonde,” “gray,” “hair” (T7)		322 (0.4)		“Gun,” “rifle,” “shooting” (T10)			232 (0.4)	
	Top 8	“Friendships,” “acquaintances,” “friends” (T9)		302 (0.4)		“Cook,” “recipes,” “cookbook” (T8)			230 (0.4)	
	Top 9	“Wedding,” “ceremony,” “bride” (T6)		295 (0.4)		“Blonde,” “gray,” “hair” (T7)			223 (0.4)	
	Top 10	“Movie,” “theater,” “film” (T13)		283 (0.3)		“Wedding,” “ceremony,” “bride” (T6)			206 (0.3)	
**Professional and financial**										
	Top 1	“Tickets,” “ticketing,” “queue” (T3)		345 (0.5)		“Lawyer,” “attorney,” “legal” (T0)			326 (0.8)	
	Top 2	“Lawyer,” “attorney,” “legal” (T0)		302 (0.5)		“Retire,” “retirement,” “enjoying” (T1)			312 (0.7)	
	Top 3	“Wear,” “dress,” “suit” (T4)		263 (0.4)		“401k,” “ira,” “roth” (T2)			241 (0.6)	
	Top 4	“Retire,” “retirement,” “enjoying” (T1)		246 (0.4)		“Security,” “social,” “collect” (T26)			193 (0.5)	
	Top 5	“Tip,” “tipping,” “delivery” (T6)		238 (0.4)		“Donate,” “charity,” “charities” (T5)			181 (0.4)	
	Top 6	“401k,” “ira,” “roth” (T2)		229 (0.4)		“Wear,” “dress,” “suit” (T4)			164 (0.4)	
	Top 7	“Name,” “named,” “nickname” (T7)		229 (0.4)		“Women,” “gender,” “men” (T14)			157 (0.4)	
	Top 8	“Paid,” “pay,” “skeptic” (T9)		206 (0.3)		“Covid,” “precovid,” “lockdown” (T8)			148 (0.3)	
	Top 9	“Interview,” “interviews,” “interviewed” (T12)		205 (0.3)		“Fly,” “airline,” “pilot” (T10)			148 (0.3)	
	Top 10	“Covid,” “precovid,” “lockdown” (T8)		204 (0.3)		“Paid,” “pay,” “skeptic” (T9)			141 (0.3)	

^a^Percentages represent the distribution among sentences successfully assigned to a theme, excluding unassigned outlier sentences (topic -1).

## Discussion

### Principal Findings

This study investigated how self-identified older Reddit users share personal experiences across 3 domains (ie, health and wellness, personal relationships and identity, and professional and financial life) and found that age-group differences emerged both at the domain level and within specific topics. The central analytic contribution is not merely that the 2 groups discuss different things but that the nature of those differences points to distinct underlying forces: life course position most powerfully structures sharing in health and professional domains, while age cohort socialization more visibly shapes expression in the relationships and identity domain. Meanwhile, pseudonymous platform affordances function as an enabling condition across all 3 domains, facilitating disclosures that identity-based networks structurally discourage. Taken together, these patterns suggest that older adults’ online self-expression cannot be adequately explained by any single framework; rather, it reflects the simultaneous, domain-specific interplay among aging, cohort, and platform.

### Health and Professional Domains: Life Course Position as the Primary Driver

The sharpest between-group contrasts in health and professional content are best understood as reflections of where each cohort stands in the life course. In health and wellness, the topics that distinguish the 2 groups correspond closely to the physiological and institutional realities of their respective life stages: hormonal change, depression, and nursing interactions for those in their 50s; Parkinson disease, dental decline, and aging awareness for those in their 60s and 70s. This pattern is consistent with life course theory’s premise that age-related transitions bring distinct social and bodily concerns that shape what individuals perceive as meaningful to communicate [24-28]. Crucially, neither group’s health discourse is simply about illness; both articulate experiences of bodily negotiation, that is, managing a changing body and its social consequences. What differs is the temporal orientation: future older adults write from a position of midlife adjustment and medical navigation, while current older adults write from one of chronic management and embodied constraint. This temporal dimension suggests that health-related sharing on Reddit may serve less as information exchange and more as a form of life-stage sense-making.

A parallel logic applies to professional and financial content. Future older adults’ emphasis on job interviews, workplace roles, and consumer transactions reflects continued embeddedness in the labor market, whereas current older adults’ focus on Social Security, pensions, and charitable giving reflects the financial and moral landscape of postretirement life. Notably, the shift from employment-centered to giving-centered discourse among current older adults is not merely a change in topic. It signals a reorientation of economic identity from productivity to stewardship. This aligns with life course research on the transition from midcareer to later adulthood, in which individuals increasingly frame financial decisions through a lens of legacy and responsibility rather than accumulation [29-32]. The presence of this discourse on a pseudonymous platform is itself significant: it implies that Reddit serves not only as a space for practical information-seeking but also as a venue for working through identity transitions that are rarely voiced in face-to-face settings.

### Relationships and Identity: Cohort Culture as the Primary Driver

If life course position explains health and professional differences, it is less adequate for understanding the divergences in personal relationships and identity. Their expressive content in this domain differs in ways that point more directly to age cohort socialization, which includes the enduring cultural habits, values, and media landscapes formed during each cohort’s formative years [33]. Future older adults’ more frequent discussions of friendships and film suggest that this cohort’s sense of self is significantly organized around shared cultural memory and relational intimacy. This cohort, consisting of Generation X, came of age during an era of expanded media consumption and shifting norms around personal expressiveness, and these formative experiences appear to persist as frameworks for identity and connection in midlife [34]. Current older adults’ greater emphasis on cooking and gun ownership is analytically distinct: rather than nostalgia or relational affirmation, these topics function as articulations of responsibility, continuity, and moral selfhood. Research on boomer generational culture documents a persistent orientation toward family stewardship and civic identity that was instilled during early socialization and continues to anchor self-narratives in later life [35-37]. On Reddit, these values are not merely expressed; they are performed for an anonymous audience, suggesting that even in pseudonymous spaces, generational identity remains a meaningful resource for self-presentation.

The cross-group similarities in this domain are equally important analytically. Both groups engaged substantively with race and LGBTQ+ identity, topics that carry heightened social risk for older cohorts in offline contexts. This convergence challenges assumptions that older adults’ online engagement is primarily conservative or identity-confirming, and points instead to Reddit’s capacity to elicit discursive engagement with contested social identities across age lines. This finding is better explained by platform affordance than by either life course or generational factors, which sets up the argument in the following section.

### Pseudonymity as an Enabling Condition for Authentic Self-Expression

Across all 3 domains, the patterns of sharing observed in this study are not fully intelligible without accounting for Reddit’s pseudonymous architecture. Pseudonymity lowers inhibitions and restructures the environment of self-disclosure, enabling users to share content that would carry social risk in identity-bound contexts [38,39]. This mechanism is most visible in health-related sharing in this study. Both age cohort groups disclosed experiences involving vulnerability, such as mental health struggles, bodily decline, and stigmatized conditions, topics that research consistently shows to be suppressed in face-to-face interactions and on real-name platforms due to stigma, self-restraint norms, and the fear of burdening others [40,41]. The willingness to share such content on Reddit is not simply a function of digital confidence; it reflects the structural condition that pseudonymity enables disclosure without consequences for one’s offline social standing. This aligns with the conceptualization of networked privacy by De Choudhury and De [42], in which users actively calibrate visibility and intimacy through selective, context-dependent disclosure, a practice that pseudonymous platforms uniquely support.

In the relationships and identity domains, pseudonymity’s enabling role takes a different form. It expands the boundaries of moral and social expression, not only emotional disclosure. The engagement of both groups with race and LGBTQ+ identity, sensitive topics that generate social friction in many older adults’ offline communities, suggests that Reddit functions as a low-risk venue for articulating positions and experiences that would otherwise be carefully managed in identity-tied settings. This expressive expansion extends to loneliness, marital life, nostalgia, and everyday frustration—contents that are affectively authentic but socially inadmissible on platforms where one’s full social identity is visible [43,44].

Taken together, these patterns position Reddit not as a neutral medium but as a structurally distinctive space that actively reconfigures what older adults are willing to say, and to whom. The act of self-identifying with an age cohort through a flair compounds this effect as users are not merely anonymous individuals but members of a loosely assembled, voluntarily constituted community of peers—a social context that provides the dual affordance of relatability and anonymity. This combination may be especially consequential for older adults, whose offline social networks, as convoy theory predicts, are contracting precisely at the life stage when the need for resonant peer exchange may be intensifying [10,11].

### Design and Policy Implications

These findings carry implications that go beyond platform research to inform *how digital environments are designed* and *how digital inclusion is conceptualized for older populations*. The results suggest that conventional digital inclusion frameworks, which tend to center operational literacy and family connectivity via identity-based platforms, leave a critical dimension unaddressed: what might be called *expressive inclusion*—the capacity to communicate authentically within a social frame that is not constrained by one’s offline identity and relationships. The finding that pseudonymous spaces enable disclosures about mental health, physical vulnerability, and contested social identities suggests that access to such digital spaces constitutes a distinct and meaningful dimension of digital participation that identity-centric interventions cannot replace.

This has practical implications for both platform design and community-based programming. Designers of eHealth platforms and digital aging services should consider integrating pseudonymous or semianonymous communication options alongside identity-tied profiles, particularly for forums addressing sensitive topics such as mental health, midlife transitions, or chronic illness. Evidence from this study suggests that such affordances may substantially increase candor and peer engagement among older users. For digital literacy practitioners, the implication is similarly concrete: curricula could incorporate “pseudonymous navigation,” teaching older adults how to identify, evaluate, and safely participate in interest-based online communities alongside the operational and security competencies that currently dominate training programs. Equipping older adults to find resonant peer communities outside their immediate offline networks would directly address the social contraction that convoy theory associates with later life.

### Limitations

Several limitations should be acknowledged when interpreting the findings. First, the study operationalizes age groups through user-selected age-based flairs. It is important to acknowledge that these labels reflect a voluntary self-categorization within a digital space, rather than objectively verified demographic membership. This choice may also bias the sample toward Reddit users who are both more digitally confident and more comfortable identifying publicly with their age. These individuals likely represent a distinctive subset of older adults. Findings should therefore be understood as characterizing active, self-identifying older Reddit users, who are a distinctive and probably more digitally agentic subset rather than older adults in general.

A second and more fundamental limitation is the cross-sectional design, which makes it impossible to disentangle life course effects from cohort effects. The differences observed between the 50s and the 60s and 70s groups could reflect either the distinct life-stage position of each group, the different generational cultures that shaped them, or their interaction. This study treats the two explanatory frameworks as complementary rather than competing but cannot empirically adjudicate their relative contributions. Doing so would require a longitudinal or cohort-sequential design capable of tracking how the same cohort’s expressive patterns shift as it moves through subsequent life stages, and this is a methodologically demanding yet theoretically essential direction for future research.

## Abbreviations

API: application programming interface
